# Microstructure and Mechanical Properties of Low- and Medium-Carbon Si-Rich Low-Alloy Steels Processed by Austemping after Intercritical Annealing

**DOI:** 10.3390/ma15031178

**Published:** 2022-02-03

**Authors:** Xin Jia, Ting Zhao, Lin Wang, Xiaowen Sun, Yuefeng Wang, Tiansheng Wang

**Affiliations:** 1 State Key Laboratory of Metastable Materials Science and Technology, Yanshan University, Qinhuangdao 066004, China; jx30307787@163.com (X.J.); ting0505ha@163.com (T.Z.); wlin0209@163.com (L.W.); sunxiaowenw@163.com (X.S.); wangyuefeng@ysu.edu.cn (Y.W.); 2 National Engineering Research Center for Equipment and Technology of Cold Rolled Strip, Yanshan University, Qinhuangdao 066004, China

**Keywords:** low- and medium-carbon steel, intercritical annealing, austempering, microstructures, mechanical properties

## Abstract

In the present paper, the designed thermomechanical process was applied to prepare ferrite/bainite multiphase microstructures in Si-rich low-alloy steel with a carbon content of 0.33 wt.% (0.33C) and 0.21 wt.% (0.21C). The microstructures were analyzed by scanning electron microscope, transmission electron microscope, and electron backscatter diffraction, and the mechanical properties (tensile and impact properties) were tested. The results showed that, on the premise of obtaining 15 vol.% ferrite in both steels, the ferrite grains in the 0.33C steel were polygonal with an average grain size of 2.2 μm, recrystallized more completely. However, the ferrite grains in the 0.21C steel were mainly long strip-shaped with a width of 2–4 μm, and the recrystallization degree was poor. In addition, upon increasing the austempering temperature, bainite ferrite laths were formed in the 0.33C steel, and the thickness was in the range of 81–123 nm. The morphology of bainite ferrite in the 0.21C steel gradually changed from lath to granular. Upon increasing the austempering temperature, the tensile strength and yield strength of both steels increased and the elongation decreased slightly. The impact energy of the two steels showed different trends upon increasing the austempering temperature, in which the impact energy of the 0.33C steel increased, while that of the 0.21C steel decreased. This is due to the difference size of the martensite-austenite constituents in the two steels.

## 1. Introduction

With the rapid development of modern industry, higher requirements are put forward for the properties of materials [1,2]. Conventional dual-phase steel, consisting of soft ferrite and hard martensite, has been widely applied in the automobile industry thanks to its good formability [3,4,5,6,7]. However, the strength and plasticity between martensite and ferrite are quite different, thus it is easy to cause cracks in the production of complex-shaped auto parts because of poor flange formability. Saeidi and Ekrami [8] tried to use bainite instead of martensite to prepare bainite/ferrite dual-phase microstructure in 4340 steel, and the mechanical properties were tested. The experimental results showed that the bainite/ferrite dual-phase microstructure had better plasticity and impact toughness than the ferrite-martensite dual-phase microstructure. In recent years, researchers have continuously optimized bainite and ferrite microstructures to further improve the properties of bainite/ferrite steel.

Caballero et al. [9,10] proposed that the low-temperature bainite microstructure could be obtained through undercooled austenite treated by austempering at a low temperature slightly higher than the *Ms* in high-C and high-Si steel. The tensile strength of this low-temperature bainite microstructure reached 1.77–2.2 GPa, and it provided considerable plasticity and fracture toughness [11,12]. Zhao et al. [13,14] reduced the *Ms* by ausrolling in medium-carbon silicon-rich steel, and obtained a low-temperature bainite microstructure with a strength of 2223–2581 MPa. Long et al. [15] prepared low-temperature bainite microstructure by continuous annealing in medium-carbon steel. Soliman et al. [16] and Qian et al. [17] achieved the purpose of reducing *Ms* by adding alloy elements. Low-temperature bainite was also prepared in the steel with a lower carbon content, and good mechanical properties were obtained. On the other hand, the ferrite grain refinement also attracted researchers’ attention. Bhattacharya et al. [18] prepared ultrafine ferrite grain microstructure (UFFG) with grain size less than 3 µm in low-carbon microalloyed steel through warm rolling followed by rapid transformation annealing (RTA) at 800–850 °C and subcritical annealing (SCA) at 600 °C. Mukherjee et al. [19] conducted cold-rolling and subsequent intercritical annealing on a fibrous ferrite/martensite starting microstructure, and obtained ultrafine-grained DP (UFG-DP) steel with an average ferrite grain size of ~2.7 µm. Jahanara et al. [20] developed refined ferrite/pearlite microstructures after 80% cold-rolled and tempering at 600 °C, and then heated to intercritical annealing temperature to obtain ultrafine grained ferrite with size of 1.6–2.5 µm.

Beladi et al. [21] prepared low-temperature bainite/fine-grain ferrite dual-phase microstructure in Mn-Si-Mo low-alloy steel containing 0.26 wt.% C using a hydraulic-servo thermomechanical simulation test machine. The process route consisted of warm deformation of supercooled austenite followed by reheating to 650 °C and holding for 1 h; the part of the austenite was decomposed into fine-grained ferrite (average grain size ~4 μm) followed by rapid cooling to 200–400 °C. This dual-phase microstructure showed an excellent combination of strength and plasticity, but the impact toughness was not tested. In addition, the holding temperature and time had a great influence on the ferrite fraction, which required precise control.

In the previous work [22], we designed a novel process route, that is, the tempered troostite microstructure was subjected to 40% cold-rolling deformation, then intercritical annealing + austempering treatment was performed to obtain ultrafine-grain ferrite/low-temperature bainite microstructure, and both strength and toughness were improved. However, alloying elements, such as Ni and W, were added to the steel, which increased the cost. In addition, the process route was relatively complicated. In the present paper, we further optimized the previous work, using warm-rolling instead of tempering + cold rolling, and applied to C-Mn-Si-Cr-Mo low-alloy steel with a different carbon content. Furthermore, the austempering temperature extended to the below-*Ms* temperature. The difference of microstructures between the two steels under the same process design was studied, and the relationship between microstructures and mechanical properties was briefly analyzed.

## 2. Materials and Experimental Methods

The low- and medium-C steels used in this study were smelted in a vacuum induction furnace and cast into ingot with a diameter of 180 mm. The code and chemical composition are shown in Table 1. The ingot was homogenized at 1150 °C for 40 min and hot-rolled into a round bar with a diameter of 40 mm at the final rolling temperature of 850 °C. The bar was air-cooled to room temperature. Subsequently, the hot-rolled round bar was kept at 880 °C for 15 h and cooled to 400 °C in a furnace, followed by air-cooling to room temperature for annealing.

**Figure 1:**
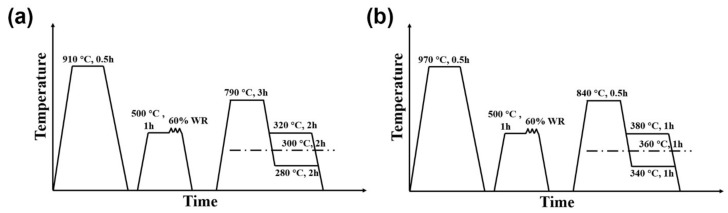
Schematic illustrations of the thermomechanical processes of the (**a**) 0.33C and (**b**) 0.21C steels.

**Figure 2:**
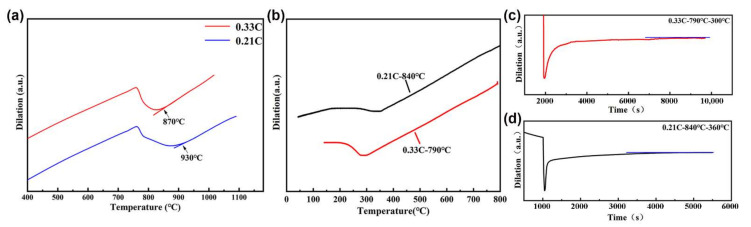
(**a**) Dilation–temperature curves to measure the *Ac_3_* temperature, (**b**) dilation–temperature curves to measure the *Ms* temperature after intercritical annealing, (**c**) dilation–time curves of the 0.33C steel, and (**d**) 0.21C steel holding at the *Ms* temperature.

The annealed round bars were machined into rectangular samples with a thickness of 30 mm, a width of 20 mm, and a length of 80 mm by wire cutting machine, and then subjected to the thermomechanical process shown in Figure 1. Firstly, the samples were heated to *Ac*_3_ + 40 °C and held for 30 min for full austenitization in a muffle furnace. The *Ac*_3_ temperatures of the 0.33C and 0.21C steels were 870 °C and 930 °C, respectively, determined by dilatometer (Netzsch, Munich, Germany), and calculated by the tangent method, as shown in Figure 2a. Subsequently, they were quenched to room temperature to obtain martensite. Secondly, the quenched samples were reheated to 500 °C and held for 1 h, followed by 60% warm-rolling deformation on rolling machine (Hengxu, Gongyi, China). The total deformation passes were nine, the reduction amount of each pass was 2 mm, and the samples were kept at 500 °C for 2 min after every three rolling passes. The final thickness of the samples was 12 mm. Finally, the deformed samples were subjected to intercritical annealing in a muffle furnace (Kaiheng, Tianjin, China) followed by austempering at *Ms* and *Ms* ± 20 °C in a salt bath furnace (potassium nitrate and sodium nitrite with a weight ratio of 1:1) (Kaiheng, Tianjin, China). The intercritical annealing temperatures of the 0.33C and 0.21C steels were 790 °C and 840 °C, respectively, and both steels obtained ~15 vol.% ferrite after intercritical annealing. The volume fraction of ferrite was determined by the statistic results of SEM (Hitachi, Tokyo, Japan) microstructures. To ensure the accuracy and reliability of statistics, 20 SEM images in each state of the both steels were counted. In addition, the *Ms* temperatures of the 0.33C and 0.21C steels after intercritical annealing were 300 °C and 360 °C, respectively (Figure 2b), measured by the Gleeble-3800 thermomechanical simulator (DSI, MN, USA). Therefore, the austempering temperatures of the 0.33C steel were 280 °C, 300 °C, and 320 °C, respectively, and the holding time was 2 h (Figure 2c), while the austempering temperatures of the 0.21C steel were 340 °C, 360 °C, and 380 °C, and the holding time was 1 h (Figure 2d).

The longitudinal section of the samples after annealing, quenching, tempering before warm-rolling, and warm-rolling was observed under an optical microscope (OM, Axiovert 200MAT, Zeiss, City Munich, Germany). The microstructures of austempered samples were characterized by scanning electron microscopy (SEM, Hitachi SU-5000, Tokyo, Japan) equipped with an electron backscattered diffraction (EBSD) attachment, transmission electron microscopy (TEM, FEI Tolas F200X, Hillsboro, OR, USA), and X-ray diffraction (XRD, Rigaku D/max-2500/PC, Tokyo, Japan). The samples for OM, SEM, and XRD were mechanically ground, polished, and then corroded with 4% nitric acid alcohol solution. Sliced disks with a diameter of 3 mm were ground mechanically down to ~30 μm in thickness, followed by double-jet thinning to perforation at a voltage of 29 V. The electrolyte consisted of 7% perchloric acid in ethanol. The foils were examined using TEM at an operating voltage of 200 kV. The treatment of EBSD samples was the same as that of TEM samples. The acceleration voltage was 70 kV and the scan step was 0.1 μm for the experiment of EBSD. XRD measurements used Cu-Kα radiation; the 2θ range was 40–105°; the scanning rate was 2°/min; and the working voltage and current were 40 kV and 200 mA, respectively.

Tensile samples with a gauge length of 25 mm, a width of 6 mm, and a thickness of 2 mm were used for tensile test on a MTS810 servo-hydraulic tensile tester, and the tensile speed was 3 mm/min. The impact toughness of the U-notched standard impact samples was tested by a 300 J Charpy impact machine (Chuanbo, Jinan, China). The size of the impact samples was 10 mm × 10 mm × 55 mm. To ensure the accuracy of the test results, the tensile and impact properties of samples in each state were tested three times.

## 3. Results and Discussion

### 3.1. Microstructures

**Figure 3:**
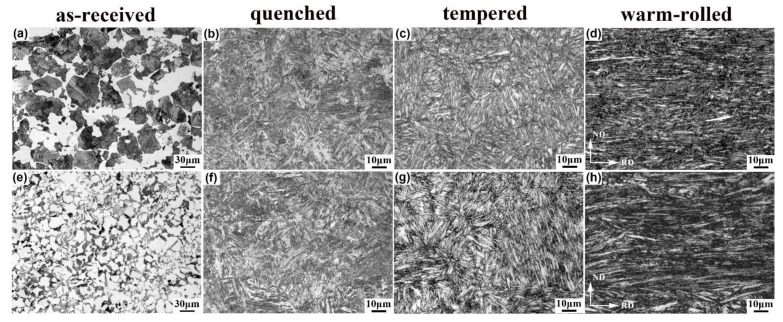
Metallographic microstructures of the (**a**–**d**) 0.33C and (**e**–**h**) 0.21C steels in different states. (**a**,**e**) Annealed; (**b**,**f**) quenched; (**c**,**g**) tempered; (**d**,**h**) warm-rolled.

Figure 3 shows the metallographic microstructures of the 0.33C and 0.21C steels in different states. It can be seen that the microstructures of the annealed samples of both steels were composed of pearlite and ferrite, and the number of pearlite in the 0.33C steel was significantly higher than that in the 0.21C steel (Figure 3a,e). The microstructures of the quenched samples of both steels were similar, which mainly were lath martensite (Figure 3b,f). After tempering at 500 °C for 1 h before warm-rolling, the morphology of the lath martensite became clear, and no carbides were precipitated (Figure 3c,g). The lath martensite tended to be aligned along the rolling direction, and the lath interfaces became blurred after warm-rolling (Figure 3d,h).

**Figure 4:**
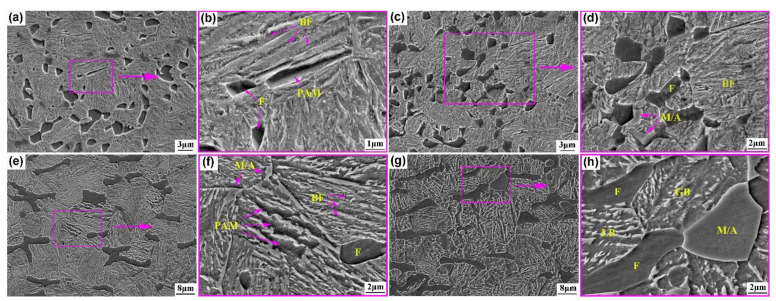
SEM images of the microstructures of the (**a**–**d**) 0.33C and (**e**–**h**) 0.21C steels austempered below *Ms* and above *Ms* after intercritical annealing. (**a**,**b**) 0.33C—280 °C; (**c**,**d**) 0.33C—320 °C; (**e**,**f**) 0.21C—340 °C; (**g**,**h**) 0.21C—380 °C.

Figure 4 shows the SEM microstructures of both steels austempered below *Ms* and above *Ms* after intercritical annealing. The fine equiaxed polygonal ferrite with a size of 1–3 μm and bainite, composed of bainite ferrite laths and film retained austenite, were obtained after austempering at different temperatures in the 0.33C steel (Figure 4a,c). After austempering at 280 °C (below-*Ms*), a certain amount of prior athermal martensite (PAM) was formed (Figure 4b). PAM resulted from some of the supercooled austenite during cooling to below-*Ms* temperature and tempered during the subsequent holding process. Some scholars have already characterized the morphology of PAM [23,24]. In addition, a small amount of M/A constituents was observed in the 0.33C sample austempered at 320 °C, which were mainly distributed at the boundaries between ferrite grains and parent austenite grains (Figure 4d). The morphologies of ferrite in the 0.21C steel were almost strip-shaped. The size ranges of long- and short-axis were 8–25 μm and 2–4 μm, respectively. The bainite ferrite laths and PAM were obtained after austempering at 340 °C, e.g., below-*Ms* (Figure 4e,f). However, after austempering at 380 °C, e.g., above-*Ms*, the amount of bainite ferrite laths decreased greatly and granular bainite was formed (Figure 4g). In addition, there existed a low content of small M/A constituents, with an average size of ~1 μm, in the 0.21C steel after austempering at 340 °C (Figure 4f). Upon increasing the austempering temperature to 380 °C, the content and size of M/A constituents increased, and the average size reached 3–6 μm (Figure 4h).

**Figure 5:**
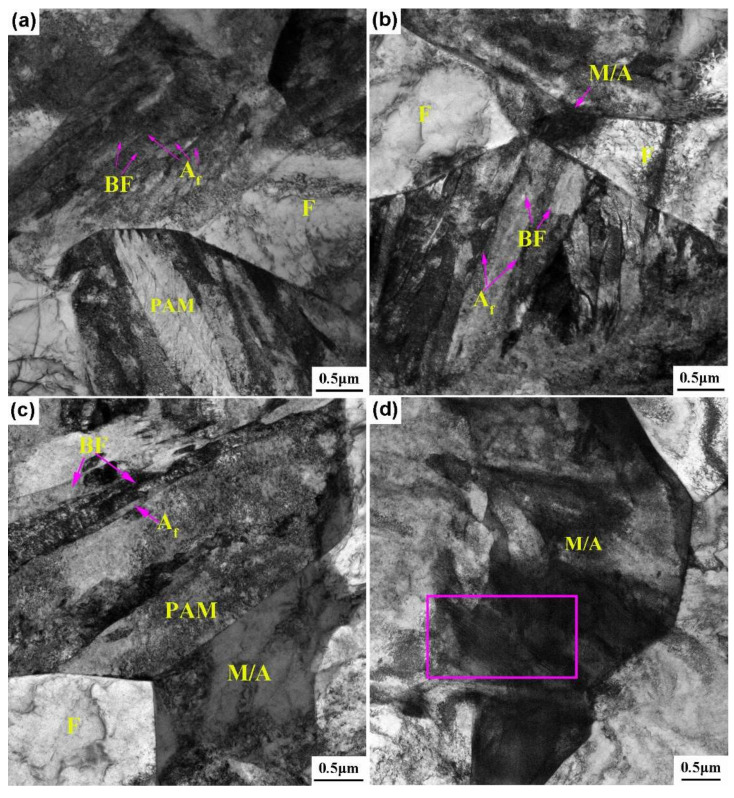
TEM microstructures of both steels after below-*Ms* and above-*Ms* austempering. (**a**) 0.33C—280 °C; (**b**) 0.33C—320 °C; (**c**) 0.21C—340 °C; (**d**) 0.21C—380 °C.

Figure 5 presents the TEM images of both steels after below-*Ms* and above-*Ms* austempering. There were many entangled dislocations in the ferrite of both steels. For the 0.33C steel, the alternately arranged bainite ferrite laths and thin-film retained austenite, and PAM were observed after austempered at 280 °C, e.g., below-*Ms* (Figure 5a). Upon increasing the austempering temperature to 320 °C, e.g., above-*Ms*, the bainite ferrite laths and thin-film retained austenite became slightly thicker, and small M/A constituents were obtained at the grain boundaries (Figure 5b). Compared with the 0.33C steel, in addition to bainite and PAM, M/A constituents were observed in the 0.21C steel after austempered at 340 °C, e.g., below-*Ms* (Figure 5c). Moreover, the size of M/A constituents obtained after austempering at 380 °C, e.g., above-*Ms*, was larger than that at 340 °C, and their existed a twin and a large number of dislocations (Figure 5d). Table 2 shows the bainite ferrite laths’ thickness *t_B_* in austempering samples of both steels. It was obtained by measuring the average linear intercept *L_T_* perpendicular to the laths’ direction and crystallographic correction with the formula *t_B_ = 2L_T_/π* [25]. It can be seen that the thickness of the bainite ferrite laths in both steels increased upon increasing the austempering temperature. The thickness of the bainite ferrite laths of the 0.33C and 0.21C steels after below-*Ms* austempering reached 80 nm and 200 nm, respectively, while the thickness after above-*Ms* austempering was 120 nm and 260 nm, respectively.

**Figure 6:**
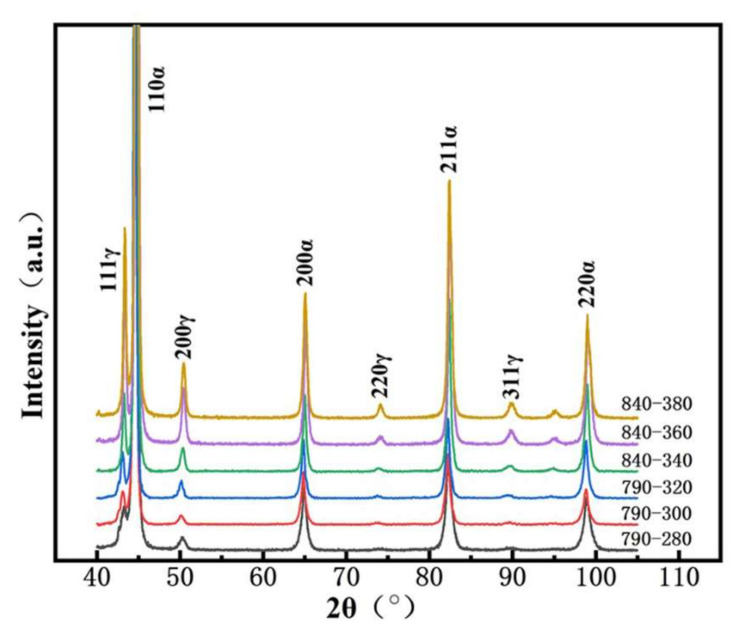
XRD patterns of the both steels austempered at different heat treatment processes.

Figure 6 displays the XRD patterns of both steels austempered at different temperatures. BCC and FCC phases were detected in both steels, corresponding to ferrite (including bainite ferrite) and retained austenite, respectively. However, no carbide was detected. The content of retained austenite (*Vγ*) was calculated from the XRD diffraction peaks and is also listed in Table 1. Upon increasing the austempering temperature, *Vγ* of both steels increased slightly, where *Vγ* of the 0.33C steel was smaller (below 9 vol.%) and *Vγ* of the 0.21C steel was higher (8.4−12.7 vol.%).

According to the observation results of the above microstructures, the ferrite content of both steels was ~15% after intercritical annealing, but there were great differences in the morphology and size of ferrite. The ferrite morphology was mainly equiaxed in the 0.33C steel, while it was mainly large long-strip in the 0.21C steel. Deformed tempered troostite microstructure, composed of ferrite matrix and a large number of dispersed fine-grained cementite, was obtained in both steels by air-cooling to room temperature after warm-rolling at 500 °C (Figure 3d,h). When deformed tempered troostite microstructure was reheated to intercritical annealing temperature, the fine-grained cementite became the nucleation site of austenite, then partial austenite was formed, and the remaining ferrite was recrystallized and refined. Therefore, the different shapes of ferrite obtained in both steels after intercritical annealing are related to the processes of ferrite recrystallization and partial austenitization during intercritical annealing. Although both of above processes accelerate upon increasing the annealing temperature, their transformation kinetics are different. Upon increasing the temperature, the thermodynamic driving force of austenite transformation and the atomic diffusion ability increase, resulting in the acceleration of transformation kinetics. However, the thermodynamic driving force of recrystallization is deformation storage energy, which does not change with temperature. Zhang et al. [26] proposed that there was a critical temperature *T_c_* in the interactive process of partial austenitization and ferrite recrystallization during intercritical annealing. When the annealing temperature was lower than *T_c_*, recrystallization occurred preferentially rather than phase transformation. The original deformed and elongated ferrite grains disappeared during recrystallization, and fine equiaxed grains were obtained. When the annealing temperature was higher than *T_c_*, phase transformation occurred preferentially, which released deformation storage energy and reduced the recrystallization driving force, while long-strip ferrite was retained. Moreover, Barbier et al. [27] proceeded with intercritical annealing at different temperatures and holding time for the steel with carbon content of 0.15 wt.% after cold-rolling deformation of 75%. The result demonstrated that only recrystallized ferrite existed in the microstructure after heating to 715 °C, followed by quenching immediately, and then the austenite increased significantly after holding at 715 °C for 1 min. However, after heating to 745 °C followed by quenching immediately, 40% of austenite and coarse anisotropic ferrite grains were already presented in the microstructure. When the treatment was finished, very long and coarse ferrite grains were retained. The experiment results showed that austenite formation and ferrite recrystallization were interacting with each other. The formation of austenite can significantly influence the kinetics of ferrite recrystallization and even inhibited it in some situations.

It was worth noting that both steels produced little or no M/A constituents after below-*Ms* austempering, indicating that the bainite transformation was more complete. This was because of the following: (1) The undercooling degree was larger after below-*Ms* austempering, which provided a greater driving force for bainite transformation [13,28]. (2) A small amount of athermal martensite was obtained after below-*Ms* austempering, and the interface between martensite and undercooled austenite provided more nucleation sites for the formation of bainite [29,30], which promoted the bainite transformation. (3) Most retained austenite existed in a stable film-shape, which was difficult to transform into an M/A constituents during air-cooling. M/A constituents were obtained in both steels after above-*Ms* austempering, but there were great differences in both the size and quantity. The parent austenite grains of the 0.33C steel were fine, the formation of blocky retained austenite was limited during austempering at 320 °C, and the thin-film retained austenite existing between bainitic ferrite laths was mainly obtained. A small amount of smaller M/A components was obtained during cooling after austempering. However, the parent austenite grains of the 0.21C steel were larger and large blocky retained austenite was obtained after austempering at 380 °C, which had a non-uniform carbon distribution. The content of carbon was lower, especially in the middle, so their stability was poor. The M/A constituents were more easily produced during cooling after austempering.

**Figure 7:**
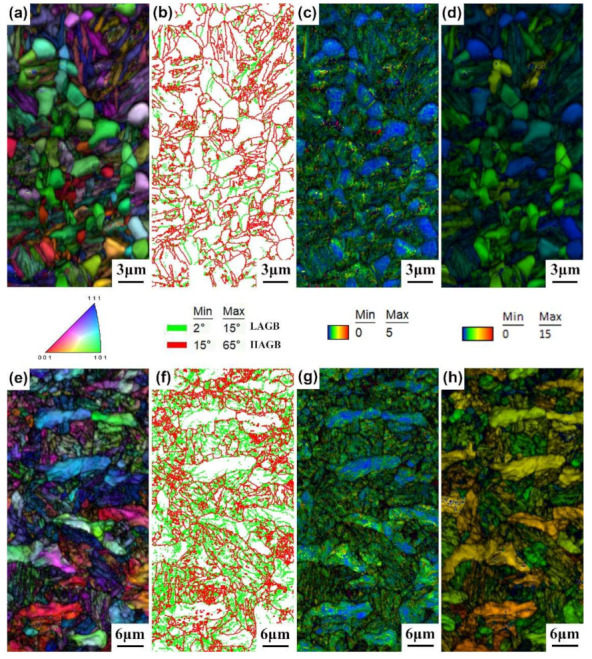
EBSD images of the (**a**–**d**) 0.33C steel and (**e**–**h**) 0.21C steel after above-*Ms* austempering. (**a**,**e**) IQ + IPF; (**b**,**f**) grain boundary distribution; (**c**,**g**) IQ + KAM; (**d**,**h**) IQ + GOS.

Figure 7 exhibits the IQ + IPF, grain boundary distribution, IQ + KAM, and IQ + GOS diagrams of the 0.33C and 0.21C steels after above-*Ms* austempering. In the grain boundary distribution diagram, red lines represent high-angle grain boundaries (15° ≤ HAGB ≤ 65°) and green lines represent low-angle grain boundaries (2° ≤ LAGB ≤ 15°). The grain orientation spread (GOS) indicates the average value of the difference between the average grain orientation and orientation of each nucleus in the grain. A smaller GOS indicates that the grain orientation gradient is lower, which means that the degree of lattice distortion is smaller. It can be seen from the IPF diagram that the color inside the per ferrite in the 0.33C steel was identical (Figure 7a), which means that the internal grain orientation is consistent; however, the color inside the grain in the large strip ferrite in the 0.21C steel was different (Figure 7e), e.g., the internal orientation of the grain is inconsistent, indicating that there is distortion inside the strip ferrite. In addition, the interfaces of the ferrite and bainite ferrite laths in both steels were mainly high-angle grain boundaries, and a small amount of low-angle grain boundaries existed at the interfaces between bainite ferrite laths (Figure 7b,f); however, compared with the 0.33C steel, the 0.21C steel had significantly more LAGBs owing to the formation of a large amount of granular bainite (Figure 7f). Furthermore, as can be observed from the KAM diagram, strain concentration occurred at the interfaces between ferrite and bainite ferrite laths and the interfaces of bainite ferrite laths in the 0.33C and 0.21C steels (Figure 7c,g). Strain concentration was also observed in the long-strip ferrite of the 0.21C steel (Figure 7g). The GOS value of equiaxed ferrite in the 0.33C steel was very low (Figure 7d), indicating that ferrite recrystallizes completely after intercritical annealing at 790 °C. The GOS value of long ferrite in the 0.21C steel was high (Figure 7h), indicating that it is still deformed ferrite and no obvious recrystallization occurs. The above microstructure results are consistent with the aforementioned analysis of the ferrite morphology and recrystallization degree.

### 3.2. Mechanical Properties

#### 3.2.1. Tensile Properties

**Figure 8:**
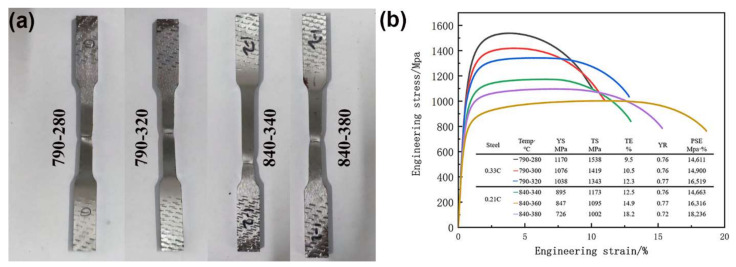
(**a**) Tensile samples after deformation and (**b**) stress–strain curves and tensile properties of both steels at different heat treatment processes.

Figure 8a,b show the tensile samples after deformation and stress–strain curves of both steels at different heat treatment processes, respectively. The tensile properties are also listed in Figure 8b. Upon increasing the austempering temperature, the tensile strength and yield strength of both steels decreased, and the elongation increased. The tensile strength of the 0.33C steel was 1343–1538 MPa, the yield strength was 1038–1170 MPa, and the elongation was 9.5–12.3%. The tensile strength of the 0.21C steel was 1002–1173 MPa, the yield strength was 726–895 MPa, and the elongation was 12.5–18.2%.

In dual-phase steel, bainite ferrite as the hard phase determines the strength. Compared with above-*Ms* austempering, both steels obtained higher tensile strength and yield strength after below-*Ms* austempering, which was due to the forming of a finer thickness of laths, resulting in the increase in strength in bainite ferrite. In addition, an obvious necking phenomenon was observed in the tensile samples after deformation of both steels (Figure 8a), which was consistent with the results in the tensile curve (Figure 8b). Compared with below-*Ms* austempering, the necking initiation of both steels after above-*Ms* austempering was delayed. This was because more retained austenite was obtained after above-*Ms* austempering, and the TRIP effect was more obvious [28,31], resulting in the delayed necking. Similarly, under the same deformation conditions, compared with 0.33C steel, 0.21C steel had more retained austenite, and necking was further delayed.

#### 3.2.2. Impact Properties

**Figure 9:**
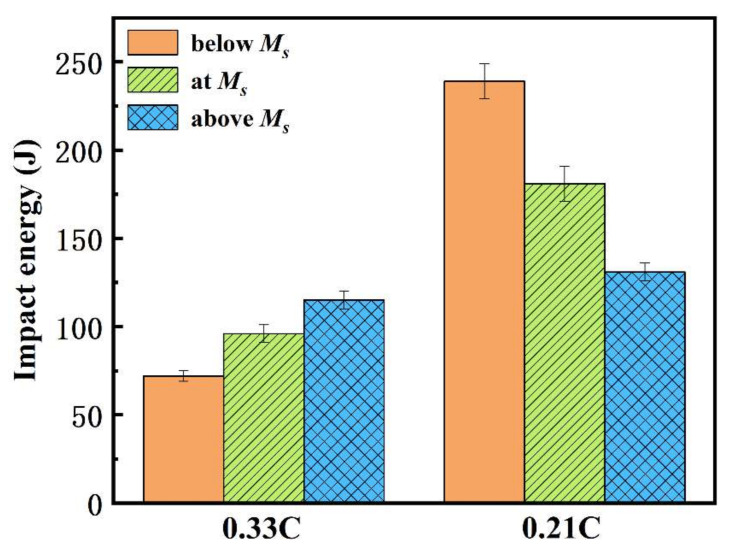
Impact energy of both steels at different heat treatment processes.

Figure 9 presents the impact energy of both steels at different heat treatment processes. For 0.33C steel, the impact energy increased upon increasing the austempering temperature, reaching 72 J and 115 J after austempering below *Ms* and above *Ms*, respectively. However, the impact energy of the 0.21C steel decreased upon increasing the austempering temperature, which was ~239 J (below-*Ms*) and 131 J (above-*Ms*). Compared with this process, the impact toughness of the 0.33C and 0.21C steels treated by the simple process without quenching and warm-rolling decreased to ~20 J and ~110 J, respectively. Based on the above microstructural observations, compared with below-*Ms* austempering, more retained austenite and M/A constituents were obtained after above-*Ms* austempering. As everyone knows, the retained austenite and M/A constituents greatly influence the impact toughness [32,33,34]. Upon increasing the austempering temperature, the content of retained austenite in the 0.33C and 0.21C steels only increased by ~2 vol.% and ~4 vol.%, respectively (Figure 8b); thus, it had only a limited influence on the impact energy. Therefore, M/A constituents are the main reason for the opposite trend of impact toughness in the two steels.

The influence of M/A constituents on impact energy is always the focus of debate. Some scholars believed that M/A constituents damage impact toughness [35], while others thought that M/A constituents can also play a positive role in impact toughness [36]. Lan et al. [37] prepared a low carbon bainite steel with microstructures of acicular ferrite, lath bainite, and M/A constituents through different heat treatment processes, and observed the SEM micrograph of the main crack surface and the secondary cracks. It can be seen that a crack originated from a large M/A constituents, and the direction of crack propagation did not change when encountering M/A constituents with the size of 5 μm. However, the crack changed its path in the vicinity of small M/A constituents with the size of less than 1 μm. Meng et al. [28] also reported a similar phenomenon in low-C high-Al/Si carbide-free bainitic Steel. Therefore, it can be clearly seen that the cracks can easily originate along the interface of the large M/A constituents. However, the small M/A constituents, especially those of size less than 1 μm, can effectively inhibit the propagation of the crack. For the present paper, the size of the M/A constituents was less than 1 μm after above-*Ms* austempering in the 0.33C steel (Figure 4d), thus the impact toughness improved. Meanwhile, the size of the M/A constituents was larger, reaching 3–6 μm after above-*Ms* austempering in the 0.21C steel (Figure 4h), resulting in a decrease in impact toughness.

#### 3.2.3. Fractographs of Impacted Samples

**Figure 10:**
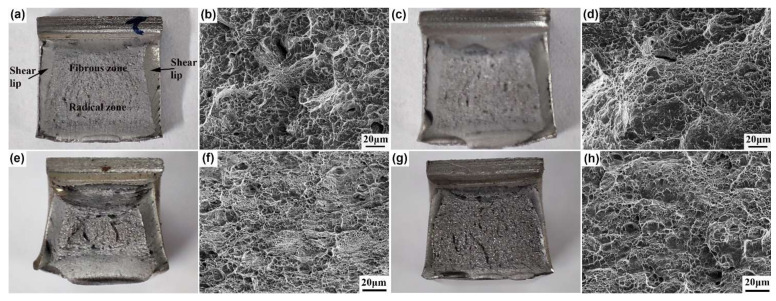
Fractographs of impacted specimens of both steels at different heat treatment processes. (**a**,**b**): 0.33C—280 °C; (**c**,**d**): 0.33C—320 °C; (**e**,**f**): 0.21C—340 °C; (**g**,**h**): 0.21C—380 °C.

Figure 10 shows the macrofractographs and microfractographs of the impact samples of both steels at different heat treatment processes. The macrofractograph mainly includes the fibrous zone, radical zone, and shear lip zone, as shown in Figure 10a. For the 0.33C steel, the difference in the macrofractographs between below-*Ms* and above-*Ms* austempering was not significant (Figure 10a,c). The microfractographs mainly included a large number of quasi-cleavage facets, and only a small amount of shallow dimples existed at the tearing ridges (Figure 10b,d), showing the characteristics of brittle fracture. For the 0.21C steel, compared with above-*Ms* austempering (Figure 10g), the shear lip zone after below-*Ms* austempering was significantly larger (Figure 10e). A large number of small dimples were observed in the microfractograph after below-*Ms* austempering (Figure 10f). The quantity of dimples obtained after above-*Ms* austempering decreased, and the size of dimples grew slightly (Figure 10h), showing the characteristics of ductile fracture.

## 4. Conclusions

(1) Fine-grain polygonal ferrite with an average grain size of 2.2 μm and bainite ferrite laths with a thickness of 81–123 nm were obtained in the 0.33C steel. However, the ferrite was mainly strip-shaped with width of 2–4 μm in the 0.21C steel, and the morphology of bainite ferrite changed from lath to lath + granular mixed morphology upon increasing the austempering temperature.
(2) The bainite transformation below *Ms* was complete, resulting in the formation of very few M/A constituents. Moreover, after above-*Ms* austempering, fine M/A constituents with the size less than 1 μm were obtained in the 0.33C steel; on the contrary, large M/A constituents with the size of 3–6 μm were obtained in the 0.21C steel.
(3) Upon increasing the austempering temperature, the strength of both steels decreased, while their elongation increased. The tensile strength of the 0.33C steel was 1343–1538 MPa, the yield strength was 1038–1170 MPa, and the elongation was 9.5–12.3%. The tensile strength of the 0.21C steel was 1002–1173 MPa, the yield strength was 726–895 MPa, and the elongation was 12.5–18.2%. However, the impact energy showed the opposite trend. Upon increasing the austempering temperature, 0.33C steel increased from 72 J to 115 J, while 0.21C steel decreased from 239 J to 131 J. This is because the fine M/A constituents in the 0.33C steel were conducive to the improvement in impact toughness, while the large M/A constituents in the 0.21C steel seriously damaged the impact toughness.

## Figures and Tables

**Table 1:** Chemical composition of both steels (wt.%).

Code	C	Mn	Si	Mo	Cr	P	S
0.33C	0.33	0.74	1.55	0.20	0.51	0.002	0.006
0.21C	0.21	0.94	1.63	0.20	0.51	0.001	0.006

**Table 2:** Bainite ferrite laths thickness *t_B_* and the content of retained austenite (*Vγ*) in austempering samples of both steels.

	0.33C			0.21C		
	790–280	790–300	790–320	840–340	840–360	840–380
Thickness of BF plates, nm	81 ± 6	98 ± 13	123 ± 18	201 ± 23	233 ± 26	264 ± 31
Volume fraction of RA, vol. %	6.2 ± 0.2	6.8 ± 0.3	8.5 ± 0.5	8.5 ± 0.3	9.5 ± 0.4	12.7 ± 0.6

## Data Availability

The raw/processed data required to reproduce these findings cannot be shared at this time as the data also form part of an ongoing study.
